# Characteristics of COVID-19-Related Deaths Among Older Adults in Malaysia

**DOI:** 10.21315/mjms2021.28.4.14

**Published:** 2021-08-26

**Authors:** Hazwan Mat Din, Raja Nurzatul Efah Raja Adnan, Siti Aisyah Nor Akahbar, Siti Anom Ahmad

**Affiliations:** 1 Malaysian Research Institute on Ageing, Universiti Putra Malaysia, Serdang, Selangor, Malaysia; 2 Department of Electrical and Electronic Engineering, Faculty of Engineering, Universiti Putra Malaysia, Serdang, Selangor, Malaysia

**Keywords:** older person, elderly, coronavirus, mortality

## Abstract

In response to the rising number of COVID-19-related deaths among older adults in Malaysia, observation concerning COVID-19-related mortality among older adults is of urgent public health importance. This study presents a review of the COVID-19-related death cases among older adults in Malaysia. Clinical and social demographic data of death cases officially released by the Ministry of Health Malaysia were reviewed. As of 12 June 2020, 81 older adult death cases were identified and included in this study. The mean age of the death cases was 71.88 years old. Even though 79% of these cases were male, gender was not likely to be associated with mortality. A substantial difference between the prevalence of diabetes among death cases and the nationwide population indicated that diabetes was more likely to be associated with mortality. Most of the studied deaths were individuals with pre-existing medical conditions, predominantly diabetes and hypertension, and those aged 70 years old or above. The mean time from hospitalisation to death was 11.83 days. Extra focus should be given to older adults in the prevention and control of COVID-19.

## Introduction

Coronavirus disease 2019 (COVID-19) is a disease caused by a virus called severe acute respiratory syndrome coronavirus 2 (SARS-CoV-2) (1). It was first identified in Wuhan, Hubei, China, in December 2019, and it spreads rapidly to the Southeast Asian countries and, throughout the world. Based on the latest data on the global burden of disease, COVID-19 will become the third leading cause of death worldwide. COVID-19 was declared a pandemic by the World Health Organization (WHO) on 11 March 2020 and is still accelerating. To date, 216 countries, areas or territories have active infections, and there have been over 12 million positive cases and at least 560,000 confirmed deaths (2).

The first wave of infection in Malaysia was from 25 January to 15 February 2020 and mainly involved Chinese tourists and other imported cases. After 11 days without new cases, the second wave began on 27 February 2020, establishing the local transmission of COVID-19 in Malaysia (3). As the number of cases steadily increased, the government announced a movement control order (MCO), which was put into effect on 18 March 2020. The first and second COVID-19-related fatalities, which were of men aged 60 years old and 34 years old, respectively, were reported one day before the MCO began. With more than 500 cases, Malaysia was the worst hit country in Southeast Asia at that time. As of 12 June 2020, the number of deaths reached 121, 67% of whom were older adults (4). A high risk of death due to COVID-19 among older adults has also been reported in other countries. For example, the United States reported that 8 out of 10 COVID-19-related deaths were older adults (5) and China reported that 83.8% of deceased COVID-19 patients were older adults (6). To date, Italy has had the highest proportion of deaths in this age group, with 96.4% of deceased patients aged 60 years old and above (7). These statistics highlight the need to understand COVID-19 mortality among older adults (8, 9).

This pandemic has impacted billions of lives around the world, especially those of older adults (10, 11). This vulnerable group experiences a severe spectrum of infections, as reported in many studies (9, 10, 12). Older adults living in retirement communities and other institutions are at high risk of COVID-19 infection (9). A study on the severity and mortality risk factors in adult COVID-19 in-patients in Wuhan revealed that older age and underlying hypertension were significantly associated with severe COVID-19 upon admission, whereas male gender, older age, cardiac injury and hyperglycaemia were associated with death in patients with severe COVID-19 (13, 14). Younger patients presented common symptoms, such as fever, cough and dyspnoea; however, in older patients, the disease could often progress to pneumonia, lung consolidation, cytokine release syndrome, endotheliitis and coagulopathy (12). Underlying diseases, such as hypertension, diabetes, cardiovascular diseases and lung diseases, also increase the risk of fatality (15, 16). These factors have led to serious complications, including multiple organ failure and death (16).

Most fatal cases of COVID-19 in Malaysia were reported with limited data available. As older adults are at high risk of developing serious infection and dying from COVID-19 (8, 10, 17), it is important to provide the public, health professionals and the authorities with useful observations concerning mortality from COVID-19 in this population (8, 18). Therefore, this study aimed to explore the characteristics of fatal cases of COVID-19 among older adults through a review of publicly available data.

## Methods

This study conducted a public retrospective record review. This method has been used to data gather in other studies of COVID-19 (18–20). Official materials and information made available by the Ministry of Health (MOH) Malaysia were collected from a public source (https://kpkesihatan.com/). The online archive of daily media releases of the Director-General of the MOH was reviewed for COVID-19 cases from 17 March to 12 June 2020, including characteristics of reported death. Only death cases involving patients aged 60 years old and above and Malaysian citizens were included in this study. The following characteristics were extracted: age, gender, number of pre-existing medical conditions, type of pre-existing medical condition and time from hospital admission to time of death (days). If the date of admission was not reported, then the case number was used to estimate the time from hospital admission to death.

### Statistical Analysis

For each measure studied, a confidence interval (CI) for the proportion was calculated. For age, proportion and mean (standard deviation [SD], range) were calculated. The mean comparison of time from hospital admission to death with the study variables was done using an independent *t*-test (gender) and analysis of variance (ANOVA) (age groups, number of preexisting medical conditions and type of medical condition). All analyses were performed using the Statistical Package for the Social Sciences (SPSS) version 20.0. Statistical significance was set at 0.05.

## Results

As of 12 June 2020, 121 deaths due to COVID-19 had been reported in Malaysia (21). Of these deaths, four were non-citizens and 36 were younger than 60 years old. Thus, 40 cases were excluded, and 81 cases (66.9%) were retained in this study.

Patient demographics and characteristics are presented in Table 1. The mean age was 71.88 years old (*n* = 81, SD = 9.08, range = 60–96). The majority of the patients (50.6%) were aged 70 years old and above (95% CI: 39.5%–61.7%), followed by 60–64 years old (25.9%; 95% CI: 16.0%–35.8%) and 65–69 years old (23.5%; 95% CI: 13.6%–33.3%). Men accounted for 79.0% (95% CI: 70.4%–87.7%) of all deceased patients. The majority of deceased patients (82.7%) had at least one pre-existing medical condition; hypertension was the most common condition (50.6%; 95% CI: 39.8%–60.8%), followed by diabetes (48.1%; 95% CI: 38.3%–58.3%), heart disease (18.5%; 95% CI: 10.8%–27.3%), kidney disease (17.3%; 95% CI: 9.9%–25.8%), cancer (7.4%; 95% CI: 2.4%–13.2%) and others (9.9%; 95% CI: 3.9%–17.1%). The other type of medication reported included stroke, gout, thyroid disease, dementia, lung disease, immobility and immune system disease.

**Figure 1:**
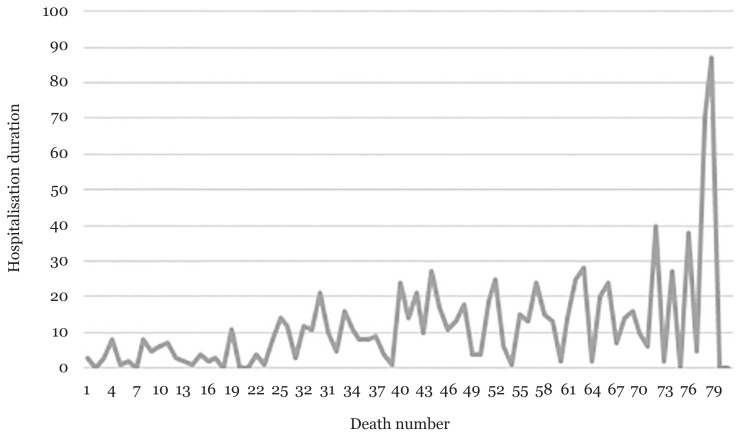
Trends of time of hospital admission to death

The time from hospital admission to death was normally distributed with a mean of 11.83 days (SD = 14.06, 95% CI: 8.72%–14.94%). Based on the mean comparison, the results revealed a non-significant *P*-value for the patient characteristics. Regarding pre-existing medical conditions, patients with other medical conditions were shown to be significantly associated with time from hospital admission to death (mean = 20.88 days; 95% CI: 14.33%–28.69%) (Table 2). When plotted against the case number, the time from hospital admission to death showed an increasing trend as the pandemic continued (Figure 1).

## Discussion

Since 12 June 2020, Malaysia has been in a positive recovery phase. However, since the disease can cause rapid, lethal infections, the country is still on high alert for the possibility of virus spread. As reported by the WHO, older adults belong to the high-risk group for COVID-19-related fatality (18). Mortality data from the Oxford COVID-19 Evidence Service indicates a risk of mortality of 3.6% for people in their 60s, which increases to 8.0% and 14.8% for people in their 70s and over 80s, respectively (11). Given this situation, an analysis of fatal cases among the older adult population has become a public health priority (11).

The study findings revealed that approximately 50% of deceased older patients were aged 70 years old or above, indicating that old age was likely to be associated with COVID-19-related mortality. A study on COVID-19 among many patients older than 60 years old also revealed a borderline significant association between age and time from initial symptoms to death, confirming that critically ill patients are more likely to be older (22, 23). In addition, a review paper revealed that the mortality rate from COVID-19 increased exponentially with age (24) and that 79% of deceased cases were men. This statistic is consistent with those of other studies reporting that men were more likely to die of COVID-19 than women (6, 25, 26). Although epidemiological data show a difference between male and female mortality rates among those diagnosed with COVID-19, the mechanisms underlying sex differences in mortality are unclear (27–29). In terms of psychological and behavioural factors, compared with women, men tend to engage in more high-risk behaviours that generate potential for contracting COVID-19 (30, 31). Moreover, compared with women, men were less likely to perform hand washing, practice physical distancing, wear masks and effectively and proactively seek medical help (30, 31).

Of the 81 deceased patients, 67 had at least one pre-existing medical condition, with hypertension (50.6%; 95% CI: 39.8%–60.8%) and diabetes (48.1%; 95% CI: 38.3%–58.3%) as the most common. As this study lacks a control group, the previous hypertension and diabetes prevalence study may elucidate the relationship between the comorbidity of these chronic diseases and COVID-19-related mortality (18). A nationwide prevalence study in 2018 indicated that 51.1% (95% CI: 48.88, 53.29) of the older adult population had hypertension and 27.7% (95% CI: 25.46, 29.99) had diabetes (32). Therefore, the substantial difference in the proportion of diabetes between the death cases (48.1%) and the older adult population (27.7%) indicated that diabetes might be associated with COVID-19-related mortality (33). However, due to the slight difference in the proportion of hypertension between the death cases (50.6%) and the older adult population (51.1%), there was insufficient evidence to suggest that hypertension was associated with COVID-19-related mortality. In sum, even though the findings suggested that only diabetes gave substantial information on the association with COVID-19-related mortality, recent studies have reported that patients with underlying diseases, such as hypertension, obesity, ischaemic heart disease, high cholesterol, kidney disease and chronic obstructive pulmonary disease, are more likely to develop severe illness or death (24, 33).

The mean comparison revealed that none of the sociodemographic variables or common chronic diseases were associated with time from hospital admission to death. Nevertheless, the increasing trend of the time from hospital admission to death has been influenced by improved treatment, medication and management of the disease, which increase the survival of the patient (34). Change of medication used to treat COVID-19 reportedly improved management of complications among older patients with multiple comorbidities (26).

Realising the immense impact of COVID-19 on older adults, the MOH and related agencies in Malaysia have taken several actions to protect this population. Information on COVID-19 and health advisories are regularly disseminated via daily press conferences, mass media and social media to increase the social responsibility of the public in ensuring the health and safety of older adults. Family members are encouraged to constantly take preventive and precautionary measures against COVID-19 as they can potentially spread COVID-19 infection to others including their older parents or grandparents (35). In addition, family members must ensure that older adults seek early treatment if they are unwell, have an adequate supply of medications and attend treatment follow-up appointments. Drive-thru and home delivery medication supply services are being provided to patients with chronic diseases to minimise exposure to the clinical/hospital environment. In addition, COVID-19 screening tests for employees and residents of public and private older adult care centres are conducted to detect positive cases and prevent further spread of infection (36).

As Malaysia is in the recovery phase of the pandemic, social distancing is still mandatory. Older adults are strongly advised to stay at home and avoid crowded places (35, 37). However, this may affect their overall physical and mental health (38). Thus, older adults must independently maintain their health by consuming nutritious food (39) and exercising regularly (40). Furthermore, they must socialise with family members and friends using technology to lessen the psychological impact of the outbreak, including loneliness, anxiety, stress and depression (41, 42).

This study presents some limitations. First, the relatively small sample size could have influenced the findings (43). For example, the number of comorbidities was likely a risk factor for mortality, albeit a not statistically significant one. Second, a control group should have been included in the study to verify the mortality risk factors identified. The lack of a control group restricts the accurate estimation of mortality (18). Finally, the study data were obtained from the official webpages of the MOH, and it is possible that other cases were made public in other ways. Considering these limitations and the limited data available for this study, the findings should be interpreted with caution.

## Conclusion

This study suggests that most COVID-19-related deaths among older adults occurred in individuals with at least one underlying pre-existing medical condition, especially among those aged 70 years old or above. Diabetes and hypertension were among the most common medical conditions among the deceased cases. The mean time from hospitalisation to death was 11.83 days. Age and underlying diseases (hypertension, diabetes, etc.) were found to be the most important risk factors for COVID-19-related deaths among older adults. Overall, more care should be given to older adults for the prevention and control of COVID-19.

## Figures and Tables

**Table 1:** Characteristic of deceased patients (*n* = 81)

Characteristics		Frequency (%)	95% CI
Mean age (SD, range)	71.88 (9.08, 60–96)		
Age	60–64	21 (25.9)	16.0, 35.8
	65–69	19 (23.5)	13.6, 33.3
	≥ 70	41 (50.6)	39.5, 61.7
Gender	Male	64 (79.0)	70.4, 87.7
	Female	17 (21.0)	12.3, 29.6
Number of pre-existing medical condition	None	14 (17.3)	8.6, 25.9
	1	25 (30.9)	21.0, 40.7
	2	20 (24.7)	14.8, 34.6
	≥ 3	22 (27.2)	17.3, 37.0
Medical condition	Diabetes	39 (48.1)	38.3, 58.3
	Hypertension	41 (50.6)	39.8, 60.8
	Kidney disease	14 (17.3)	9.9, 25.8
	Heart disease	15 (18.5)	10.8, 27.3
	Cancer	6 (7.4)	2.4, 13.2
	Others	8 (9.9)	3.9, 17.1

**Table 2:** Comparison between time of hospital admission to death (days) and characteristics of deceased patients

Characteristics		Time of hospital admission to death (days)		

		Mean (95% CI)	*t*/F	*P*
Age (years old)	60–64	13.57 (7.16, 22.78)	0.26	Not significant
	65–69	12.00 (7.88, 16.17)		
	≥ 70	10.85 (7.39, 15.68)		
Gender	Male	11.84 (9.07, 15.35)	0.02	Not significant
	Female	11.76 (4.14, 21.89)		
Number of pre-existing medical condition	None	8.86 (2.44, 20.49)	0.73	Not significant
	1	10.04 (7.00, 13.25)		
	2	12.65 (5.70, 22.46)		
	≥ 3	15.00 (10.50, 19.45)		
Medical condition	Diabetes	13.31 (9.27, 18.54)	−0.91	Not significant
	Hypertension	12.90 (9.72, 16.30)	−0.69	Not significant
	Kidney disease	17.71 (8.50, 31.52)	−1.74	Not significant
	Heart disease	11.93 (7.67, 16.54)	−0.03	Not significant
	Cancer	4.67 (0.80, 10.00)	1.30	Not significant
	Others	20.88 (14.33, 28.69)	−1.95	Significant
